# Association of blood urea nitrogen to albumin ratio with cerebral small vessel disease and its ischemic imaging markers: a cross-sectional study

**DOI:** 10.3389/fneur.2026.1763901

**Published:** 2026-05-21

**Authors:** Qian Luo, Jieying Zhuang, Huijuan Wang, Ruiyan Xiao, Xudong Yang, Xiangkun Fu, Shujun Hong, Huizhen Weng, Jiangping Cai

**Affiliations:** 1 Department of Neurology, The First Hospital of Quanzhou Affiliated to Fujian Medical University, Quanzhou, China; 2 The Graduate School of Fujian Medical University, Fuzhou, China; 3 The School of Clinical Medicine, Fujian Medical University, Fuzhou, China

**Keywords:** blood urea nitrogen/albumin ratio, cerebral small vessel disease, cross-sectional study, ischemic imaging markers, mechanism

## Abstract

**Background:**

This study aims to assess the association between blood urea nitrogen to albumin ratio (BAR) and cerebral small vessel disease (CSVD) and its common ischemic neuroimaging markers, including white matter hyperintensities (WMH), enlarged perivascular spaces (EPVS), and lacunes.

**Methods:**

This cross-sectional study involved 762 participants, including 452 patients with CSVD and 310 non-CSVD controls. Their clinical data, hematological markers, and neuroimaging results were collected. The relationship between the BAR index and CSVD/ischemic neuroimaging markers was assessed using Spearman’s rank correlation coefficient, multivariable logistic regression, and restricted cubic splines. Subgroup analyses were performed stratified by various covariates. Model performance and calibration were evaluated using ROC analysis, bootstrap validation, and calibration curves. Sensitivity analyses were conducted to assess the robustness of associations against varying levels of kidney function.

**Results:**

After adjusting for confounding factors, BAR was positively associated with CSVD (OR: 1.77, 95% CI: 1.35–2.33, *p* < 0.001), WMH (OR:1.55, 95% CI: 1.22–1.96, *p* < 0.001), and EPVS (OR: 2.09, 95% CI: 1.71–2.57, *p* < 0.001). However, BAR was not related to lacunes (OR: 1.12, 95% CI: 0.92–1.36, *p* = 0.302). In BAR’s classification model, compared to the lowest quantile, the OR for the risk of CSVD in the middle and highest quantiles was 2.40 (95% CI: 1.68–3.43) and 4.25 (95% CI: 2.91–6.21), respectively (P for Trend < 0.001). In restricted cubic splines, after adjusting covariates, the association between BAR and CSVD (P_overall_ < 0.001, P_non-linear_ = 0.872) and WMH (P_overall_ = 0.001, P_non-linear_ = 0.145) was linear and dose-responsive, while the relationship was nonlinear with EPVS (P_non-linear_ = 0.001). Crucially, sensitivity analyses confirmed the robustness of these associations against varying degrees of kidney function. Additionally, ROC analysis revealed that BAR was associated with CSVD (AUC: 0.701, 95% CI: 0.664–0.738). Model 4 displayed the optimal discriminative performance (AUC: 0.914, 95% CI: 0.895–0.933), which was further supported by bootstrap internal verification and excellent calibration (Brier score = 0.117).

**Conclusion:**

BAR is independently associated with CSVD and its ischemic imaging markers, highlighting its potential as a novel and cost-effective biomarker. These findings warrant further investigation to determine its utility for risk stratification and its potential role in primary care screening protocols.

## 1 Introduction

Cerebral small vessel disease (CSVD) refers to a group of pathological processes affecting cerebral arterioles, venules, and capillaries, with various etiologies and characterized by clinical, neuroimaging, and neuropathological manifestations (1). Its incidence is positively correlated with age, affecting approximately 5% of individuals over 50 years of age and nearly 100% of those over 90 years of age (2). The STRIVE-2 report summarizes the imaging markers associated with CSVD from a neuroimaging perspective (3), including ischemic markers such as lacunes, white matter hyperintensities (WMH), and enlarged perivascular spaces (EPVS), as well as hemorrhagic markers like cerebral microbleeds (4). The clinical manifestations of CSVD are highly heterogeneous, ranging from stroke and progressive cognitive impairment to motor and mood disorders, ultimately leading to a loss of independence and increased mortality (5–8). Since the early symptoms of CSVD are often non-specific and the disease can be clinically silent in its early phases, the current diagnosis of CSVD predominantly relies on neuroimaging (3, 9, 10), which underscores the critical need for complementary, accessible biomarkers to facilitate early identification and risk stratification.

The pursuit of such laboratory markers has led to the investigation of systemic processes linked to CSVD. Notably, the imaging features of CSVD, such as WMH, cerebral microbleeds, and EPVS, are associated with markers of kidney function decline (11, 12). Blood urea nitrogen (BUN), the end product of nitrogen metabolism and a key marker of kidney function, remains underexplored in relation to CSVD, despite its established role in predicting mortality in systemic vascular conditions like acute coronary syndrome (13). Furthermore, serum albumin, synthesized in the liver, provides insights into liver and kidney function as well as the body’s nutritional status (14). Several studies (15, 16) have demonstrated that serum albumin is vital for maintaining physiological homeostasis, including endothelial stability, oxidant scavenging, normal colloid osmotic pressure, and the transport of endogenous compounds. From a functional perspective, there appears to be a relationship between serum albumin and CSVD (17), supported by evidence linking low albumin levels to an increased risk of ischemic stroke and larger WMH volumes (18, 19).

Critically, the blood urea nitrogen to albumin ratio (BAR) is a composite index that integrates two distinct pathways of systemic compromise: kidney function and metabolic stress (reflected by BUN) with systemic inflammation, endothelial vulnerability, and nutritional status (reflected by albumin). This ratio has been established as a prognostic marker in various systemic conditions, such as pneumonia (20), gastrointestinal bleeding in the elderly (21), sepsis (22), chronic obstructive pulmonary disease (23), COVID-19 (22), and cardiovascular disease (24). Notably, a large-scale retrospective study has demonstrated that the BAR serves as a novel biomarker for discriminating cardiovascular disease, cardiovascular mortality, and all-cause mortality in individuals with diabetes (25). Furthermore, a cross-sectional study has shown that BAR is associated with specific neuroimaging markers, including WMH, lacunes, and cerebral microbleeds (26). Nonetheless, a more comprehensive investigation is warranted to determine whether the composite index BAR provides a stronger association with CSVD than its individual components, a question that remains unanswered.

To address this gap, this cross-sectional study comprehensively assessed the association between BAR and CSVD, as well as its common ischemic imaging markers (lacunes, WMH, and EPVS). A key objective is to determine whether the composite index BAR offers enhanced utility for identifying CSVD compared to the separate evaluation of BUN and albumin. Our findings may inform the future development of efficient diagnostic strategies for CSVD.

## 2 Methods

### 2.1 Participants and ethics

This study is a cross-sectional study. The inpatients and outpatients from January 1, 2020, to January 1, 2024, in the Neurology Department of The First Hospital of Quanzhou Affiliated to Fujian Medical University were recruited. Inclusion criteria: (1) Age of participants ranged from 30 to 85 years. (2) Patients underwent examination using a 3.0 T MRI scanner, including T1-weighted imaging (T1WI), T2-weighted imaging (T2WI), fluid-attenuated inversion recovery imaging (FLAIR), and diffusion-weighted imaging (DWI). (3) Patients received hematological examinations, which included routine blood tests and biochemical markers such as serum creatinine and serum albumin. (4) Patients had complete clinical data available. Exclusion criteria: (1) Patients with acute cerebral infarction, with lesions exhibiting high signals on the DWI phase and a diameter greater than 20 mm. (2) Patients with acute cerebral hemorrhage. (3) Patients with acute subarachnoid hemorrhage, a history of cerebral vascular malformation or aneurysmal subarachnoid hemorrhage, or the identification of an untreated aneurysm (diameter > 3 mm). (4) Patients with neurodegenerative diseases such as Alzheimer’s disease or Parkinson’s disease. (5) Patients with definitive non-vascular white matter lesions, including multiple sclerosis, adult white matter dysplasia, and metabolic encephalopathy. (6) Patients with mental illness diagnosed according to DSM-V diagnostic criteria. (7) Presence of contraindications for MRI examinations (e.g., claustrophobia). (8) Patients classified as moderately or severely malnourished (body mass index (BMI) < 16.9 kg/m^2^). (9) Patients with serious organic diseases, including advanced liver disease, patients undergoing hemodialysis or receiving human albumin infusion, or patients with hematological disorders, autoimmune diseases, chronic kidney diseases (serum creatinine > 1.5 mg/dL), chronic immunodeficiency (e.g., patients with HIV, those undergoing chemotherapy, or taking prednisolone or other immunosuppressants), chronic obstructive pulmonary disease, sepsis, massive gastrointestinal bleeding, or severe heart failure. (10) Patients with incomplete clinical data. Fasting blood samples for laboratory analysis, including BAR, were obtained during the initial clinical evaluation upon hospital admission. This preceded the brain MRI examination and the initiation of any major, non-emergent treatment regimens, thereby establishing a consistent temporal sequence from exposure assessment to outcome ascertainment. This study was approved by the Ethics Committee of the First Hospital of Quanzhou (Approval number: [2025]K239) and performed in accordance with the Declaration of Helsinki. The participants provided their written informed consent to participate in this study.

### 2.2 Data collection

Baseline demographic and clinical data were collected by two well-trained investigators. The collected baseline data included gender, age, height, weight, systolic blood pressure (SBP), diastolic blood pressure (DBP), and history of smoking, drinking, hypertension, diabetes, coronary heart disease, and stroke. All subjects’ heights and weights were measured using standardized procedures. Height was measured in meters (m) to two decimal places, while weight was measured in kilograms (kg) to one decimal place. The BMI was calculated as weight (kg) divided by height (m^2^) and rounded to two decimal places. Smoking history was defined according to World Health Organization (WHO) criteria as having smoked continuously or cumulatively for 6 months or more during one’s lifetime. Drinking history was assessed per WHO standards, where men consuming over 20 grams per occasion and women consuming over 10 grams per occasion, at least twice a week for a minimum of 1 year, were classified as regular drinkers. Hypertension was defined as having blood pressure measurements of ≥140/90 mmHg on three or more occasions in 1 day without antihypertensive medication, or having a previous diagnosis of hypertension requiring treatment. Diabetes was diagnosed based on the presence of typical symptoms (such as polydipsia, polyuria, polyphagia, and unexplained weight loss) alongside a fasting blood glucose level of ≥7.0 mmol/L, a random blood glucose level of ≥11.1 mmol/L, or a two-hour oral glucose tolerance test result of ≥11.1 mmol/L. Alternatively, individuals without typical symptoms could be diagnosed if they had blood glucose levels exceeding three standards on two or more occasions in 1 day, or if they had a prior diagnosis of diabetes and were currently using hypoglycemic medications. Coronary heart disease was identified through definitive electrocardiogram changes or diagnostic records from past evaluations. Stroke history was established based on identifiable imaging changes or diagnostic records indicating a prior stroke diagnosis.

### 2.3 Laboratory evaluation

Peripheral blood samples were collected after a fasting period of at least 8 h. The examination items included white blood cell count, neutrophil count, lymphocyte count, C-reactive protein (CRP), total cholesterol (TC), low-density lipoprotein cholesterol (LDL-C), high-density lipoprotein cholesterol (HDL-C), albumin, fasting plasma glucose (FPG), glycosylated hemoglobin (HbA1c), estimated glomerular filtration rate (eGFR), uric acid, creatinine, and urea nitrogen. As previously described (26–28), the BAR, which is a unitless index, was calculated using the formula: BAR = BUN (mg/dL) / albumin (g/dL). The reference ranges in our clinical laboratory were 8–21 mg/dL for BUN and 4.0–5.5 g/dL for albumin.

### 2.4 MRI evaluation

The MRI evaluations were conducted using a 3.0 T MRI scanner (Signa, GE Healthcare, Milwaukee, WI, United States). The brain MRI images were analyzed by two neuroimaging experts who were blinded to clinical baseline characteristics and laboratory data. Inter-rater reliability for the categorical MRI markers was excellent, as indicated by Cohen’s kappa (*κ*) statistics: for overall CSVD diagnosis, *κ* = 0.865 (95% confidence intervals (CI): 0.828–0.902); for WMH burden grade, κ = 0.858(95% CI: 0.819–0.893); for EPVS burden grade, κ = 0.845 (95% CI: 0.806–0.884); and for lacunes, κ = 0.861 (95% CI: 0.822–0.900); all *p* values < 0.001. Three imaging markers of CSVD related to ischemic pathophysiology: WMH, EPVS, and lacunes, were assessed. Representative images for each severity grade of these markers are provided in Supplementary Figure S1. WMH exhibited high signal intensity on T2WI and FLAIR, while presenting low signal intensity on T1WI. Using a semi-quantitative approach, WMH was graded according to the Fazekas scoring system (29), with scores ranging from 0 to 3, assessing periventricular WMH (PWMH) and deep WMH (DWMH) separately. The scoring criteria for PWMH were as follows: 0 = no lesions, 1 = cap-shaped or pencil-shaped thin-layer lesions, 2 = smooth halo lesions, and 3 = irregular high signals near the ventricle extending into the deep white matter. The DWMH scoring standard was: 0 = no lesions, 1 = punctate lesions, 2 = lesions beginning to merge, and 3 = a large confluent area lesion. The overall WMH burden was calculated as the sum of PWMH and DWMH scores, resulting in a total range of 0 to 6 points. According to the severity of the total WMH burden, the study population was categorized into a mild WMH group (total WMH score of 0–2 points) and a moderate-to-severe WMH group (WMH score of 3–6 points).

On MRI, lacunes presented as low signal on T1WI, high signal on T2WI, and a surrounding high signal ring on FLAIR, appearing as round or oval lesions with a diameter of 3–20 mm. Lacunes were classified based on their number (0 or ≥1).

EPVS showed low signals on T1WI and FLAIR sequences, while displaying high signals on T2WI sequences, with either small dots (diameter < 3 mm) or linear high signals on T2WI images. A quantitative method was employed, collecting extensive data from the bilateral cerebral hemispheres and categorizing them by count. The classification was as follows: none (0 EPVS), grade 1 (1–10 EPVS), grade 2 (11–20 EPVS), grade 3 (21–40 EPVS), and grade 4 (> 40 EPVS) (30). For classification convenience, the EPVS burden was divided into mild (0–1) and moderate (2–4).

### 2.5 Definition of composite CSVD outcome

For the primary binary outcome of this study, participants were classified as having ‘CSVD present’ if their brain MRI and clinical profile met at least one of the following criteria: (1) Moderate-to-severe WMH, defined as a total Fazekas score ≥ 2; (2) Mild WMH (total Fazekas score = 1) in the presence of more than two vascular risk factors (hypertension, hyperlipidemia, diabetes mellitus, obesity, or smoking); (3) Mild WMH (total Fazekas score = 1) co-existing with one or more lacunes; and, (4) Presence of a new subcortical lacunar infarction with a diameter of less than 20 mm on MRI. This composite diagnostic framework was adapted from the enrollment criteria of the China Imaging-based Biobank of Cerebral Small Vessel Diseases cohort study, as previously described and utilized in studies of Asian populations with CSVD (31). The neuroimaging evaluation of all CSVD markers adhered to the definitions outlined in the Standards for Reporting Vascular Changes on Neuroimaging (STRIVE) (32). Furthermore, to ensure a rigorously defined non-CSVD control group, participants were confirmed to be free of any of the nine core imaging markers of CSVD as comprehensively described in the STRIVE-2 criteria (3), including recent small subcortical infarcts, lacunes, WMH, EPVS, cerebral microbleeds, cortical superficial siderosis, cortical cerebral microinfarcts, brain atrophy, and incidental DWI-positive lesions. Participants who did not meet any of the CSVD criteria and exhibited none of these nine imaging markers were classified as the non-CSVD control group.

### 2.6 Statistical analysis

All statistical analyses and graphical productions were conducted using R software (version 4.4.3) and GraphPad Prism 10 (GraphPad Software Inc.). Two-tailed tests were employed, and statistical significance was determined at a *p*-value of less than 0.05. Missing data were excluded from the study. The Kolmogorov–Smirnov test was utilized to assess the normality of numerical variables. Continuous normally distributed variables are represented by the mean and standard deviation, while continuous variables with non-normal distribution are characterized by the median and interquantile range. Categorical variables are expressed as proportions. The Mann–Whitney U test, Chi-squared (χ^2^) test, or Fisher’s exact test was employed to evaluate statistical differences in clinical data between the two groups. Spearman’s correlation was used to assess the relationship between covariates, CSVD, and common markers.

To avoid multicollinearity, variables with a variance inflation factor (VIF) > 10 were excluded from the multivariate model (33). After variable selection, all retained covariates had VIFs < 10, including clinically correlated pairs such as hypertension with SBP/DBP, diabetes with fasting glucose/HbA1c, and dyslipidaemia history with lipid measurements, confirming acceptable collinearity. Subsequently, a multivariate model, incorporating both categorical and continuous variables, was used to analyze the relationship between BAR and CSVD, as well as common imaging markers of ischemic CSVD. In the classification model, the BAR values were divided into three quantiles, using the median of each quantile as a continuous variable to assess linear trends. Additionally, four sequential models were constructed to progressively adjust for potential confounders: Model 1 (unadjusted), Model 2 (adjusted for sex and age), Model 3 (adjusted for sex, age, BMI, SBP, DBP, smoking status, and history of drinking, hypertension, diabetes, coronary heart disease, and stroke), and Model 4 (further adjusted for lymphocyte count, CRP, TC, LDL-C, HDL-C, FPG, HbA1c, and uric acid). This staged approach moves from demographic factors to established vascular risk factors (medical history and blood pressure) and finally to laboratory-based metabolic and inflammatory markers, allowing assessment of how each successive level of adjustment influences the BAR-CSVD association.

The dose–response relationship between BAR levels and CSVD, along with its ischemic neuroimaging markers, was analyzed using the restricted cubic splines (RCS). Additionally, patients were stratified into subgroups based on sex, age, hypertension, smoking status, FPG, BMI, LDL-C, HDL-C, and uric acid. Interaction tests were conducted to assess the association between BAR and CSVD across different subgroups. To verify the model performance, receiver operating characteristic (ROC) curves were plotted. The area under the ROC curve (AUC) was calculated, and the optimal cutoff values were identified. The bootstrap method using 1,000 bootstrap samples was conducted for internal validation. The calibration of the discriminative models (BAR alone (Model 1) and the full multivariate Model 4) was assessed to evaluate the agreement between the predicted and observed probabilities of CSVD, using quantitative metrics including the calibration slope, intercept, and Brier score.

Sensitivity analyses were performed to assess the influence of kidney function on the BAR-CSVD association: (1) excluding patients with eGFR < 60 mL/min/1.73 m^2^ (*n* = 43); (2) re-including patients with mild kidney impairment (serum creatinine 1.5–2.0 mg/dL; *n* = 18) who had been excluded under the original criterion of creatinine > 1.5 mg/dL; and (3) incorporating eGFR as an additional covariate in Model 4. Although creatinine > 1.5 mg/dL was an exclusion criterion, residual confounding by kidney function may persist within the normal range; forcibly adjusting for eGFR in this sensitivity analysis serves to assess whether the BAR-CSVD association is independent of even subtle variations in kidney function.

## 3 Results

### 3.1 Clinical and demographic characteristics of participants

**Figure 1:**
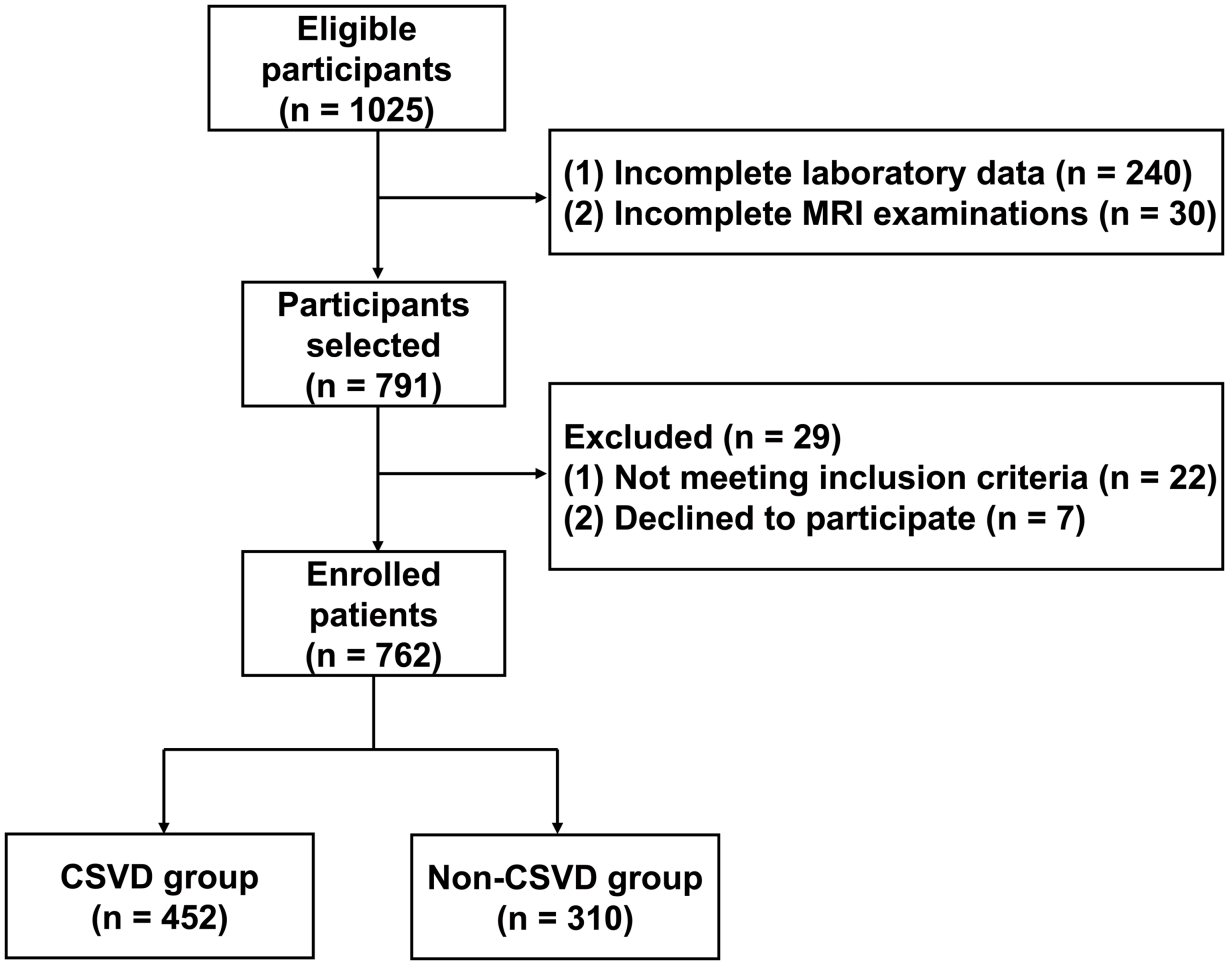
The flowchart of patient enrollment.

**Figure 2:**
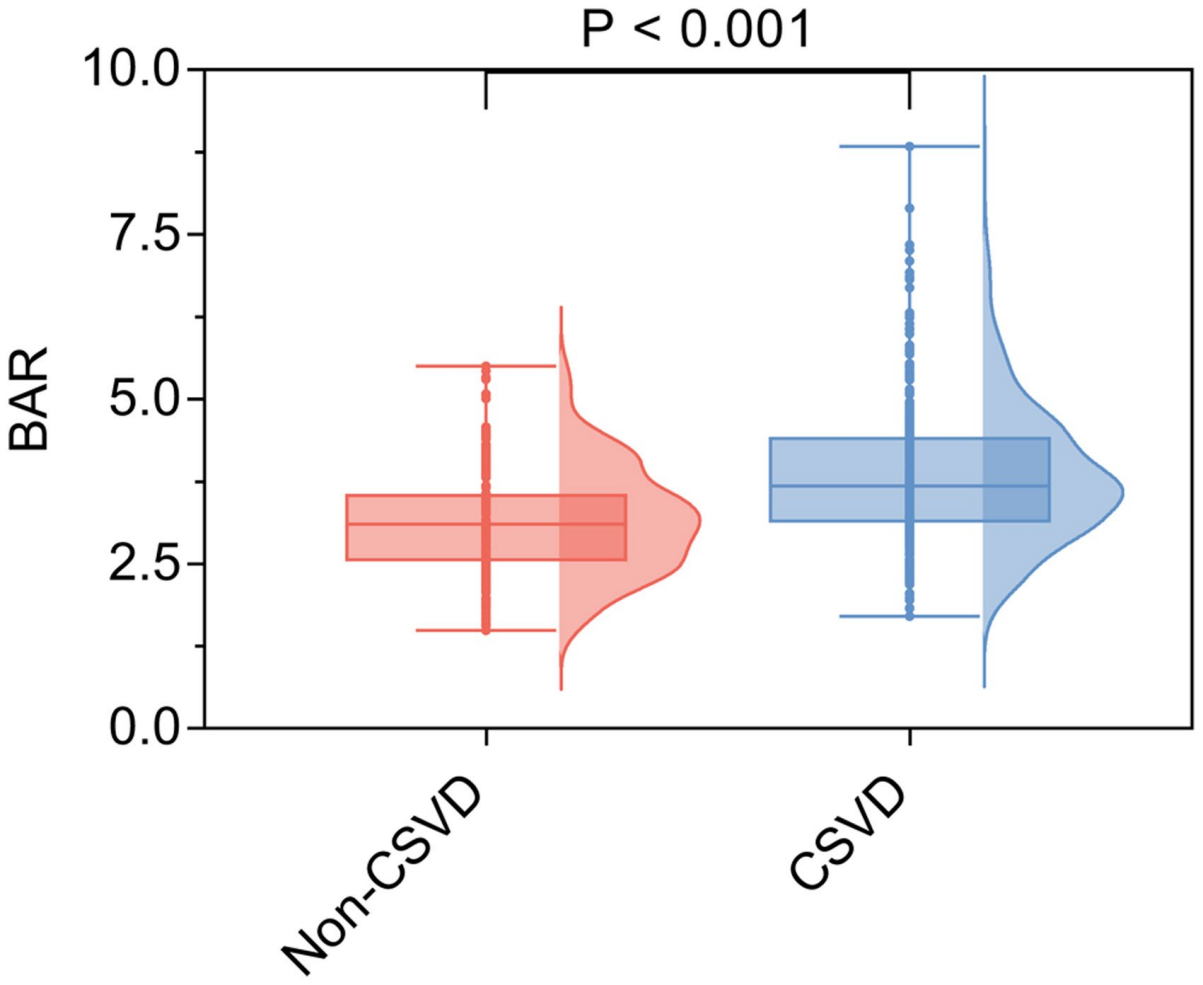
Comparison of the BAR level between the CSVD group and the non-CSVD group. The bar graph illustrates the median BAR with interquartile range in patients with CSVD compared to non-CSVD controls. The BAR was significantly higher in the CSVD group (*p* < 0.001, Mann–Whitney *U* test).

A total of 762 participants were enrolled in this study (Figure 1). Their demographic characteristics and clinical data are presented in Table 1. Among these, 310 patients were categorized into the non-CSVD group and 452 patients into the CSVD group. There was no statistically significant difference in BMI between the CSVD group and the non-CSVD group (*p* > 0.05). Significant differences were noted in the variables, including age, sex, SBP, DBP, history of smoking, drinking, hypertension, diabetes, coronary heart disease, and stroke, white blood cell count, lymphocyte count, CRP, TC, LDL-C, HDL-C, FPG, HbA1c, eGFR, creatinine, urea nitrogen, and albumin (*p* < 0.05). Notably, the level of BAR in the CSVD group was significantly higher than that in the non-CSVD group (*p* < 0.001; Figure 2). The distribution and overlap of the imaging markers within the CSVD group are detailed in Table 2. The majority of patients (85.39%) qualified based on the presence of moderate-to-severe WMH. Substantial overlap was observed among the imaging markers; for instance, among those with moderate-to-severe WMH, 47.57% also had moderate-to-severe EPVS, and an identical proportion had lacunes.

**Table 1:** Characteristics of the study population.

Variables	Total (*n* = 762)	Non-CSVD (*n* = 310)	CSVD (*n* = 452)	*p*
Age (years)	63.00 (52.00, 70.00)	53.00 (46.00, 62.00)	67.00 (61.00, 73.00)	<0.001
Male (%)	403 (52.89)	125 (40.32)	278 (61.50)	<0.001
BMI (kg/m^2^)	23.48 (21.59, 24.91)	23.35 (21.48, 25.29)	23.53 (21.77, 24.78)	0.816
SBP (mmHg)	138.00 (123.00, 156.00)	125.00 (115.25, 140.00)	146.00 (131.00, 165.00)	<0.001
DBP (mmHg)	84.00 (76.00, 95.00)	80.00 (73.00, 89.00)	86.50 (78.00, 98.00)	<0.001
Smoking, n (%)	218 (28.22)	42 (13.55)	173 (38.27)	<0.001
Drinking, n (%)	93 (12.20)	13 (4.19)	80 (17.70)	<0.001
Hypertension, n (%)	380 (49.87)	78 (25.16)	302 (66.81)	<0.001
Diabetes, n (%)	114 (14.96)	12 (3.87)	102 (22.57)	<0.001
History of CHD, n (%)	55 (7.22)	6 (1.94)	49 (10.84)	<0.001
History of stroke, n (%)	104 (13.65)	5 (1.61)	99 (21.90)	<0.001
WBC (10^9^/L)	7.14 (5.79, 8.67)	6.81 (5.70, 8.38)	7.28 (5.87, 8.83)	0.033
Neutrophil count (10^9^/L)	4.30 (3.30, 5.92)	4.02 (3.12, 5.53)	4.58 (3.48, 6.08)	0.001
Lymphocyte count (10^9^/L)	1.78 (1.40, 2.25)	1.92 (1.52, 2.39)	1.70 (1.31, 2.10)	<0.001
CRP (mg/L)	1.72 (0.50, 4.07)	0.58 (0.50, 2.50)	2.25 (0.52, 5.00)	<0.001
TC (mmol/L)	4.88 (4.13, 5.70)	5.00 (4.32, 5.99)	4.80 (4.04, 5.51)	<0.001
LDL-C (mmol/L)	3.12 (2.51, 3.76)	3.24 (2.69, 3.88)	3.04 (2.42, 3.67)	<0.001
HDL-C (mmol/L)	1.13 (0.98, 1.36)	1.17 (1.02, 1.42)	1.11 (0.94, 1.33)	<0.001
FPG (mmol/L)	5.21 (4.75, 5.93)	5.04 (4.65, 5.53)	5.42 (4.83, 6.29)	<0.001
HbA1c (%)	5.80 (5.54, 6.30)	5.62 (5.40, 5.90)	6.00 (5.70, 6.60)	<0.001
eGFR (mL/min × 1.73 m^2^)	94.82 (82.01, 105.81)	102.09 (91.06, 111.73)	88.31 (75.59, 100.20)	<0.001
Uric acid (μmol/L)	333.00 (274.00,404.75)	313.50 (268.00,386.50)	349.00 (281.75,418.00)	<0.001
Creatinine (μmol/L)	67.60 (57.40, 81.30)	62.50 (52.85, 74.77)	71.35 (60.77, 84.45)	<0.001
Blood urea nitrogen (mg/dL)	13.58 (11.42, 15.98)	12.63 (10.29, 15.14)	14.28 (12.21, 16.92)	<0.001
Albumin (g/dL)	3.95 ± 0.36	4.07 ± 0.33	3.86 ± 0.36	<0.001
BAR	3.49 (2.93, 4.12)	3.10 (2.54, 3.56)	3.68 (3.13, 4.43)	<0.001

CSVD, cerebral small vessel disease; BMI, body mass index; SBP, systolic blood pressure; DBP, diastolic blood pressure; CHD, coronary heart disease history; WBC, white blood cell; CRP, C-reactive protein; TC, total cholesterol; LDL-C, low-density lipoprotein cholesterol; HDL-C, high-density lipoprotein cholesterol; FPG, fasting plasma glucose; HbA1c, glycosylated hemoglobin; eGFR, estimated glomerular filtration rate; BAR, Blood urea nitrogen to serum albumin ratio.

**Table 2:** Classification and imaging marker overlap in the CSVD group (*n* = 452).

Qualification path and marker overlap	*n* (%)
Qualified by moderate-to-severe WMH (Fazekas ≥2)	386 (85.39%)
Among them, with moderate-to-severe EPVS	215 (47.57%)
Among them, with lacunes	215 (47.57%)
Qualified by mild WMH + risk factors	39 (8.63%)
Among them, with moderate-to-severe EPVS	18 (3.98%)
Among them, with lacunes	16 (3.54%)
Qualified by mild WMH + Lacune(s), without risk factors	3 (0.66%)
Among them, with moderate-to-severe EPVS	1 (0.22%)
Qualified by a new lacunar infarction	24 (5.31%)
Among them, with moderate-to-severe EPVS	10 (2.21%)
Among them, with confluent WMH (Fazekas ≥2)	0 (0%)

CSVD, cerebral small vessel disease; WMH, white matter hyperintensities; EPVS, enlarged perivascular spaces.

### 3.2 Correlation analysis between clinical features and the risk of CSVD

**Figure 3:**
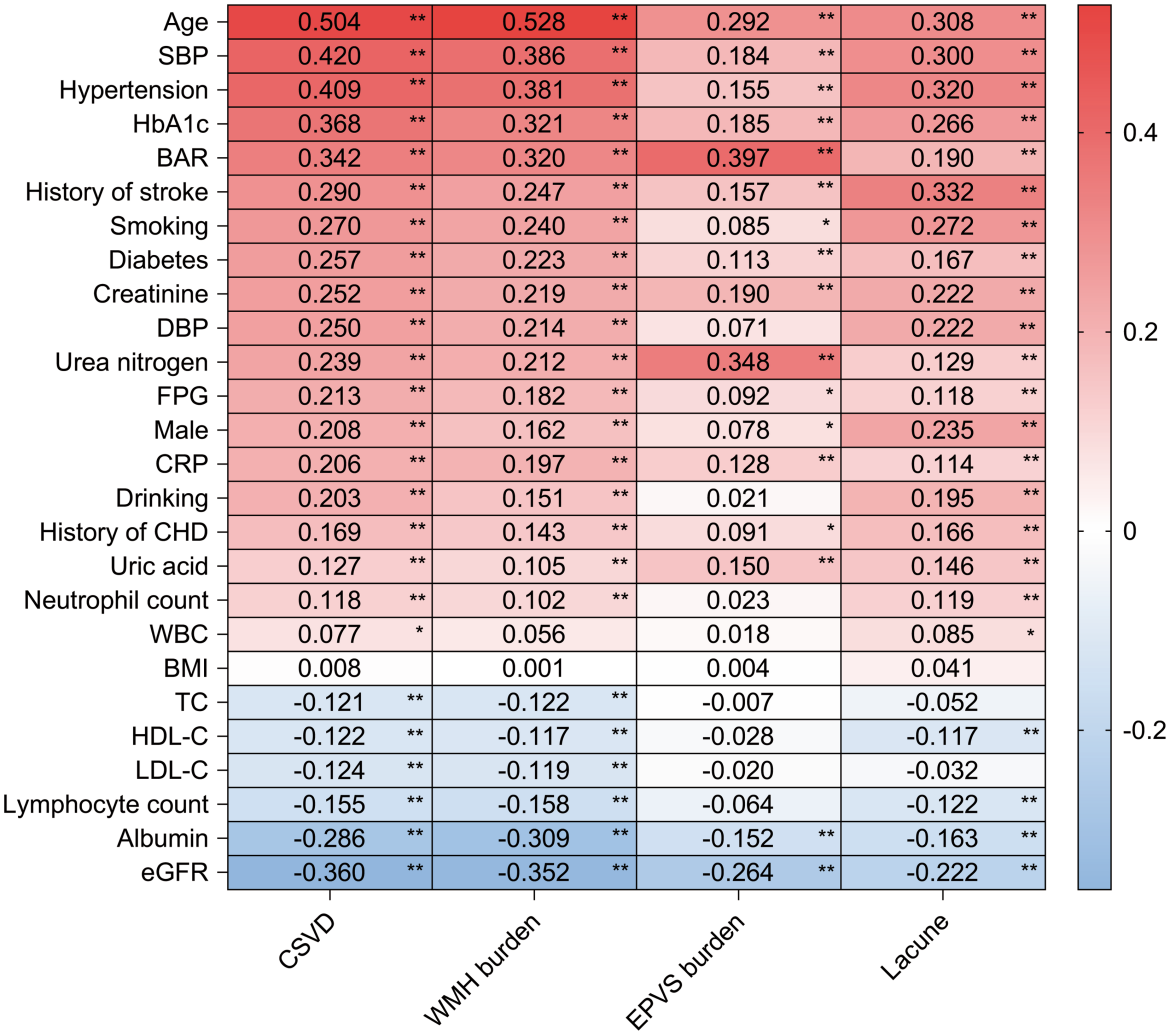
Spearman correlation between clinical features, CSVD, and its ischemic imaging markers. Correlation matrix depicting the strength and direction of associations between various clinical features and the risk of CSVD, including its ischemic imaging markers (lacunes, WMH, and EPVS). *p < 0.05, **p < 0.01.

The Spearman correlation analysis was conducted to assess the relationship between clinical features and the risk of CSVD, along with its associated ischemic imaging markers (Figure 3). The variables of age, SBP, hypertension, HbA1c, BAR, history of stroke and diabetes, and coronary heart disease, CRP, uric acid, urea nitrogen, and creatinine were found to be positively correlated with the risk of CSVD, lacunes, WMH burden, and EPVS (*r* > 0.1, *p* < 0.001). Conversely, albumin and eGFR were negatively correlated with the risk of CSVD and its related ischemic imaging markers (*r* < −0.1, *p* < 0.001). In contrast, BMI showed no significant correlation with the risk of CSVD or its associated imaging markers (*r* = 0.008, *r* = 0.0001; *r* = 0.004; r = 0.041; *p* > 0.05).

### 3.3 Multivariate regression analysis of the relationship between BAR and CSVD

To further investigate the relationship between BAR levels and CSVD, we constructed a multivariate regression model (Table 3). BAR levels were categorized into three quantiles with the following cut points: Quantile 1: BAR ≤ 3.076; Quantile 2: BAR 3.078–3.858; and Quantile 3: BAR > 3.859. BAR values are presented as the median of each quantile. The results indicated that patients in Quantile 3 and Quantile 2 had a 1.40-fold (odds ratio [OR]: 2.40, 95% CI: 1.68–3.43) and 3.25-fold (OR: 4.25, 95% CI: 2.91–6.21) increased risk, respectively, compared to those in Quantile 1. Although the correlation weakened after adjusting for potential confounding factors, it remained statistically significant and demonstrated a linear trend (OR: 1.44, 95% CI: 1.07–1.92, P for trend = 0.015). In the continuous variable model, the BAR value was significantly positively associated with the CSVD risk (OR: 2.37, 95% CI: 1.96–2.87, *p* < 0.001). These associations remained significant even after adjusting for potential confounding factors (OR: 1.77, 95% CI: 1.35–2.33, *p* < 0.001).

**Table 3:** Multivariate regression analysis of the association between BAR and CSVD risk.

Quantiles of BAR	No. of participants/incidence	Model 1	Model 2	Model 3	Model 4
		OR (95% CI)	OR (95% CI)	OR (95% CI)	OR (95% CI)
Quantile 1 (≤3.076)	762/252	1.00 (Reference)	1.00 (Reference)	1.00 (Reference)	1.00 (Reference)
Quantile 2 (3.078–3.858)	762/258	2.40 (1.68–3.43)	1.44 (0.95–2.19)	1.51 (0.92–2.47)	1.41 (0.84–2.36)
Quantile 3 (>3.859)	762/252	4.25 (2.91–6.21)	1.85 (1.19–2.88)	2.12 (1.27–3.54)	1.97 (1.14–3.392)
OR for quantile trend	762/762	2.18 (1.78–2.68)	1.39 (1.10–1.76)	1.50 (1.14–1.97)	1.44 (1.07–1.92)
P for trend		<0.001	0.006	0.004	0.015
OR for BAR value	762/762 (1.480–8.880)	2.37 (1.96–2.87)	1.65 (1.33–2.05)	1.81 (1.39–2.34)	1.77 (1.35–2.33)
*p* value		<0.001	<0.001	<0.001	<0.001

OR, Odds Ratio; CI, Confidence Interval; CSVD, cerebral small vessel disease; BAR, Blood urea nitrogen to serum albumin ratio. Model 1: Crude model without adjustment. Model 2: Adjusted for sex (male) and age. Model 3: Adjusted for sex (male), age, body mass index, systolic blood pressure, diastolic blood pressure, smoking status, and history of drinking, hypertension, diabetes, coronary heart disease, and stroke. Model 4: Adjusted for sex (male), age, body mass index, systolic blood pressure, diastolic blood pressure, smoking status, history of drinking, hypertension, diabetes, coronary heart disease, and stroke, lymphocyte count, C-reactive protein, total cholesterol, low-density lipoprotein cholesterol, high-density lipoprotein cholesterol, fasting plasma glucose, hemoglobin A1c, and uric acid levels.

### 3.4 Multivariate regression analysis of the relationship between BAR and the common imaging markers of CSVD

As shown in Table 4, multivariate regression analysis indicated that after adjusting for potential confounders of WMH burden, EPVS burden, and lacunes, the Quantile 3 of BAR exhibited significantly greater associations compared to the Quantile 1 of BAR: a 0.75-unit increase (OR: 1.75, 95% CI: 1.06–2.88) for WMH burden and a 4.06-unit increase (OR: 5.06, 95% CI: 3.24–7.90) for EPVS burden. Both associations displayed significant linear trends (P for trend = 0.025 for WMH burden and P for trend < 0.001 for EPVS burden). Conversely, no evidence was found to support a linear relationship between lacunes and BAR (OR: 1.07, 95% CI: 0.82–1.39, P for trend = 0.625). Similarly, continuous variable models adjusted for all relevant confounding factors yielded consistent results: BAR values exhibited significant positive associations with both WMH burden (OR: 1.55, 95% CI: 1.22–1.96, *p* < 0.001) and EPVS burden (OR: 2.09, 95% CI: 1.71–2.57, *p* < 0.001). Additionally, a post-hoc region-specific analysis was conducted to evaluate the association between BAR and EPVS burden in the basal ganglia and centrum semiovale separately. This analysis confirmed that higher BAR levels remained significantly associated with moderate-to-severe EPVS burden in both the basal ganglia (OR: 1.50, 95% 1.22–1.85, *p* < 0.001) and the centrum semiovale (OR: 1.46, 95% CI: 1.20–1.78, *p* < 0.001) after full adjustment. However, no statistically significant association was found between BAR values and lacunes (OR: 1.12, 95% CI: 0.92–1.36, *p* = 0.302). To further explore this null association, we tested for effect modification by clinically relevant variables known to be linked to lacunar pathophysiology, including hypertension, age, and diabetes history. In models with lacunes as the sole outcome and fully adjusted for Model 4 covariates, no significant interactions were observed between BAR and hypertension (P for interaction = 0.385), age > 65 years (P for interaction = 0.330), or diabetes history (P for interaction = 0.260).

**Table 4:** Multivariate regression analysis of the association between BAR and the common imaging markers CSVD.

Quantiles	No. of participants/incidence	Model 1	Model 2	Model 3	Model 4
		OR (95% CI)	OR (95% CI)	OR (95% CI)	OR (95% CI)
Moderate to severe WMH burden					
Quantile 1 (≤3.076)	762/252	1.00 (Reference)	1.00 (Reference)	1.00 (Reference)	1.00 (Reference)
Quantile 2 (3.078–3.858)	762/258	2.06 (1.44–2.94)	1.14 (0.75–1.74)	1.07 (0.67–1.71)	1.00 (0.62–1.63)
Quantile 3 (>3.859)	762/252	4.00 (2.76–5.79)	1.73 (1.12–2.68)	1.79 (1.11–2.90)	1.75 (1.06–2.88)
OR for quantile trend	762/762	2.10 (1.72–2.56)	1.34 (1.06–1.70)	1.37 (1.06–1.77)	1.36 (1.04–1.77)
P for trend		<0.001	0.013	0.016	0.025
OR for BAR value	762/762 (1.480–8.880)	2.17 (1.81–2.59)	1.51 (1.23–1.86)	1.55 (1.24–1.95)	1.55 (1.22–1.96)
*p* value		<0.001	<0.001	<0.001	<0.001
Moderate to severe EPVS burden					
Quantile 1 (≤3.076)	762/252	1.00 (Reference)	1.00 (Reference)	1.00 (Reference)	1.00 (Reference)
Quantile 2 (3.078–3.858)	762/258	3.84 (2.54–5.80)	3.07 (2.01–4.70)	3.07 (2.00–4.72)	2.94 (1.90–4.55)
Quantile 3 (>3.859)	762/252	7.73 (5.09–11.72)	5.53 (3.58–8.55)	5.50 (3.54–8.55)	5.06 (3.24–7.90)
OR for quantile trend	762/762	2.88 (2.32–3.57)	2.41 (1.93–3.02)	2.41 (1.92–3.03)	2.30 (1.83–2.90)
P for trend		<0.001	<0.001	<0.001	<0.001
OR for BAR value	762/762 (1.480–8.880)	2.53 (2.09–3.05)	2.19 (1.80–2.67)	2.18 (1.78–2.66)	2.09 (1.71–2.57)
*p* value		<0.001	<0.001	<0.001	<0.001
Lacune					
Quantile 1 (≤3.076)	762/252	1.00 (Reference)	1.00 (Reference)	1.00 (Reference)	1.00 (Reference)
Quantile 2 (3.078–3.858)	762/258	1.43 (0.96–2.14)	0.87 (0.56–1.35)	0.77 (0.48–1.25)	0.74 (0.45–1.21)
Quantile 3 (>3.859)	762/252	2.42 (1.64–3.57)	1.22 (0.79–1.89)	1.14 (0.71–1.85)	1.07 (0.66–1.75)
OR for quantile trend	762/762	1.61 (1.31–1.98)	1.14 (0.90–1.44)	1.10 (0.85–1.43)	1.07(0.82–1.39)
P for trend		<0.001	0.278	0.454	0.625
OR for BAR value	762/762 (1.480–8.880)	1.56 (1.33–1.82)	1.19 (1.00–1.42)	1.14 (0.94–1.38)	1.12 (0.92–1.36)
*p* value		<0.001	0.053	0.183	0.302

OR, Odds Ratio; CI, Confidence Interval; CSVD, cerebral small vessel disease; BAR, Blood urea nitrogen to serum albumin ratio. Model 1: Crude model without adjustment. Model 2: Adjusted for sex (male) and age. Model 3: Adjusted for sex (male), age, body mass index, systolic blood pressure, diastolic blood pressure, smoking status, and history of drinking, hypertension, diabetes, coronary heart disease, and stroke. Model 4: Adjusted for sex (male), age, body mass index, systolic blood pressure, diastolic blood pressure, smoking status, history of drinking, hypertension, diabetes, coronary heart disease, and stroke, lymphocyte count, C-reactive protein, total cholesterol, low-density lipoprotein cholesterol, high-density lipoprotein cholesterol, fasting plasma glucose, hemoglobin A1c, and uric acid levels.

### 3.5 The dose–response relationship between BAR and CSVD using RCS analysis

**Figure 4:**
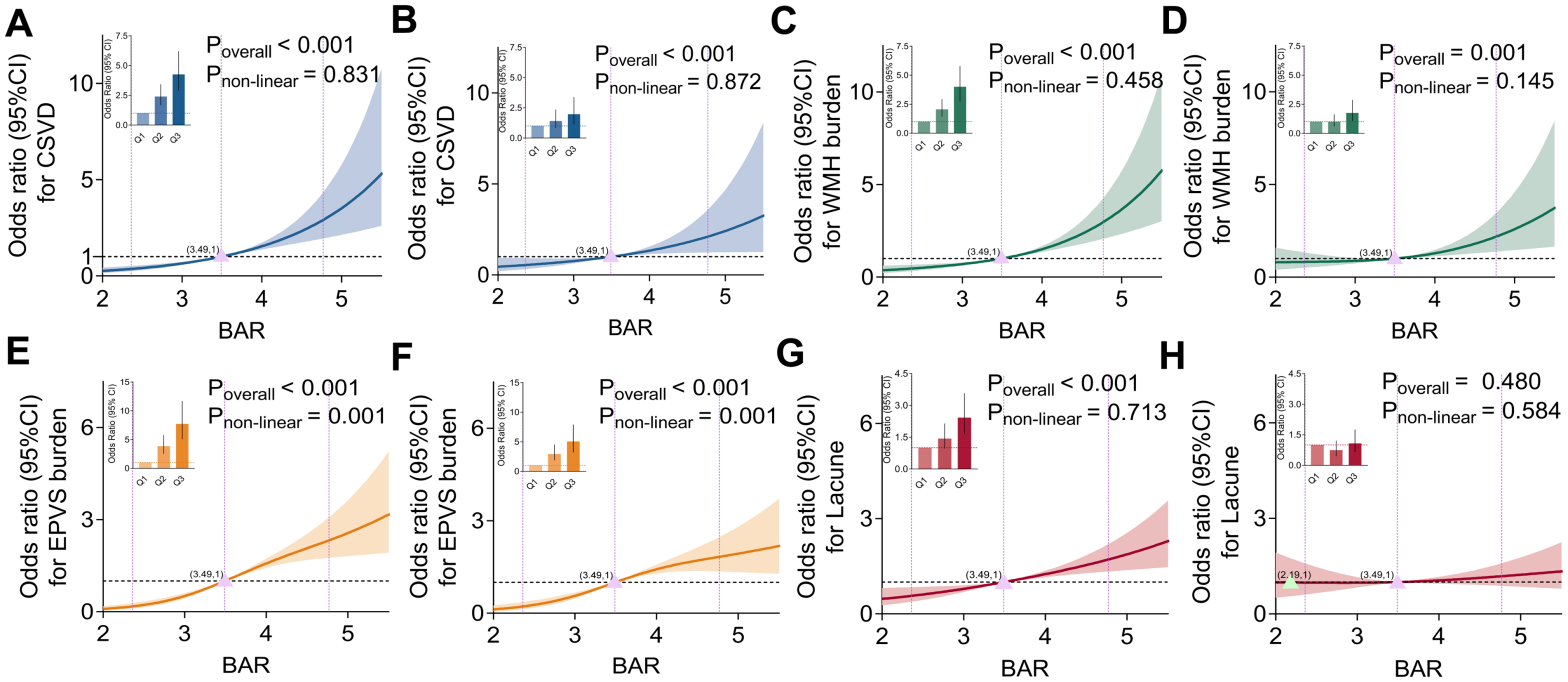
Dose–response relationships between BAR, CSVD, and its ischemic imaging biomarkers. RCS analyses were conducted to evaluate the dose–response associations between BAR and CSVD **(A,B)**, WMH **(C,D)**, EPVS **(E,F)**, and lacunes **(G,H)**. The adjusted non-linear and linear associations with relevant covariates are presented in **(B,D,F,H)**. The size and central position of the quantile bars represent the ORs, while error bars and shaded areas indicate the corresponding 95% CIs. The knots for the RCS were placed at the 10th, 50th, and 90th percentiles of the BAR distribution and are marked on the *x*-axis of each panel with vertical dashed lines. The dashed horizontal line at an OR of 1 represents the reference line. Inflection points, where the confidence band crosses OR = 1, are marked with purple and green triangles. The *p*-values for overall association (*P*overall) and non-linearity (*P*non-linear) are displayed in each panel. The relationships between the quantiles of BAR and CVSD, along with the imaging markers, are depicted in the bar plot in each panel. Quantile 1 (Q1): BAR ≤ 3.076; Quantile 2 (Q2): BAR 3.078–3.858; and Quantile 3 (Q3): BAR > 3.859.

RCS analyses were conducted to evaluate the dose–response associations between BAR and CSVD (Figures 4A,B), WMH (Figures 4C,D), EPVS (Figures 4E,F), and lacunes (Figures 4G,H). The results demonstrated that linear dose–response relationships were observed for CSVD (P for non-linearity = 0.831; Figure 4A), WMH (P for non-linearity = 0.458; Figure 4C), and lacunes (P for non-linearity = 0.713; Figure 4G). However, a significant non-linear dose–response relationship was identified between BAR and EPVS (P for non-linearity = 0.001; Figure 4E). Covariates, including sex, age, BMI, SBP, DBP, smoking status, history of drinking, hypertension, diabetes, coronary heart disease, and stroke, lymphocyte count, CRP, TC, LDL-C, HDL-C, FPG, HbA1c, and uric acid, were adjusted for in Figures 4B,D,F,H. The results indicated that the dose–response relationships were found to be linear for CSVD (P for non-linearity = 0.872; Figure 4B) and WMH (P for non-linearity = 0.145; Figure 4D), with no strong evidence supporting a non-linear association in these instances. Conversely, a significant non-linear dose–response relationship was observed between BAR and EPVS (P for non-linearity = 0.001; Figure 4F). Moreover, there was no significant association between BAR and lacunes (P for overall association = 0.480, P for non-linearity = 0.584; Figure 4H), suggesting that BAR does not exhibit a meaningful linear or non-linear relationship with lacunes in this study. Similar patterns of associations were observed for the relationships of BAR quantiles, CSVD, and its imaging markers, as depicted in the bar plot in each panel (Figures 4A–H).

### 3.6 Subgroup analysis

**Figure 5:**
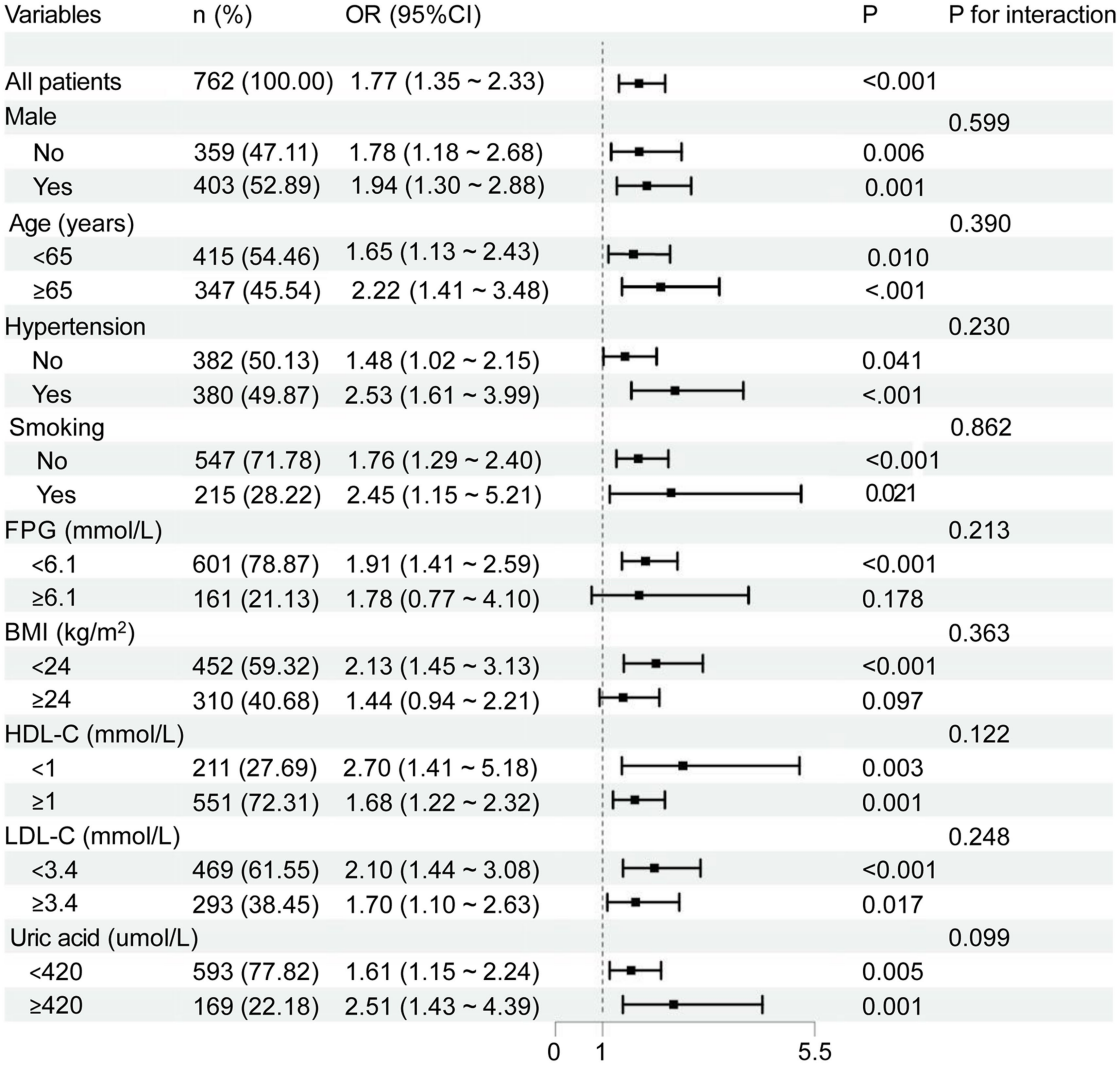
Subgroup analysis and interaction effects of the relationship between BAR and CSVD. Forest plot showing the results of multivariable logistic regression analyses assessing the association between BAR levels and the risk of CSVD across various subgroups, including sex, age, hypertension status, smoking status, FPG, BMI, LDL-C, HDL-C, and uric acid. OR, Odds ratio; CI, Confidence interval.

To further assess the association between BAR and the risk of CSVD, we conducted subgroup analyses based on sex, age, hypertension, smoking status, FPG, BMI, LDL-C, HDL-C, and uric acid. After adjusting for potential clinical confounding factors, multivariable logistic regression analysis was applied within each subgroup. The results revealed that the positive association between elevated BAR and increased CSVD risk remained consistent and statistically significant in the majority of subgroups (Figure 5). Notably, the association was not significant in participants with a BMI ≥ 24 kg/m^2^ and those with impaired fasting glucose (FPG ≥ 6.1 mmol/L). Moreover, no significant interactions were detected among the subgroup variables (all P for interaction > 0.05; Figure 5). Additionally, we formally tested for effect modification by low LDL-C levels. No significant interaction was observed, whether LDL-C was dichotomized at the clinical cutoff of 3.4 mmol/L (P for interaction = 0.248), at an alternative cutoff of 2.6 mmol/L (P for interaction = 0.872), or treated as a continuous variable (P for interaction = 0.326), indicating that the relationship between BAR and CSVD is largely independent of LDL-C status.

### 3.7 The discriminative value of BAR and models 1 to 4 for CSVD

**Figure 6:**
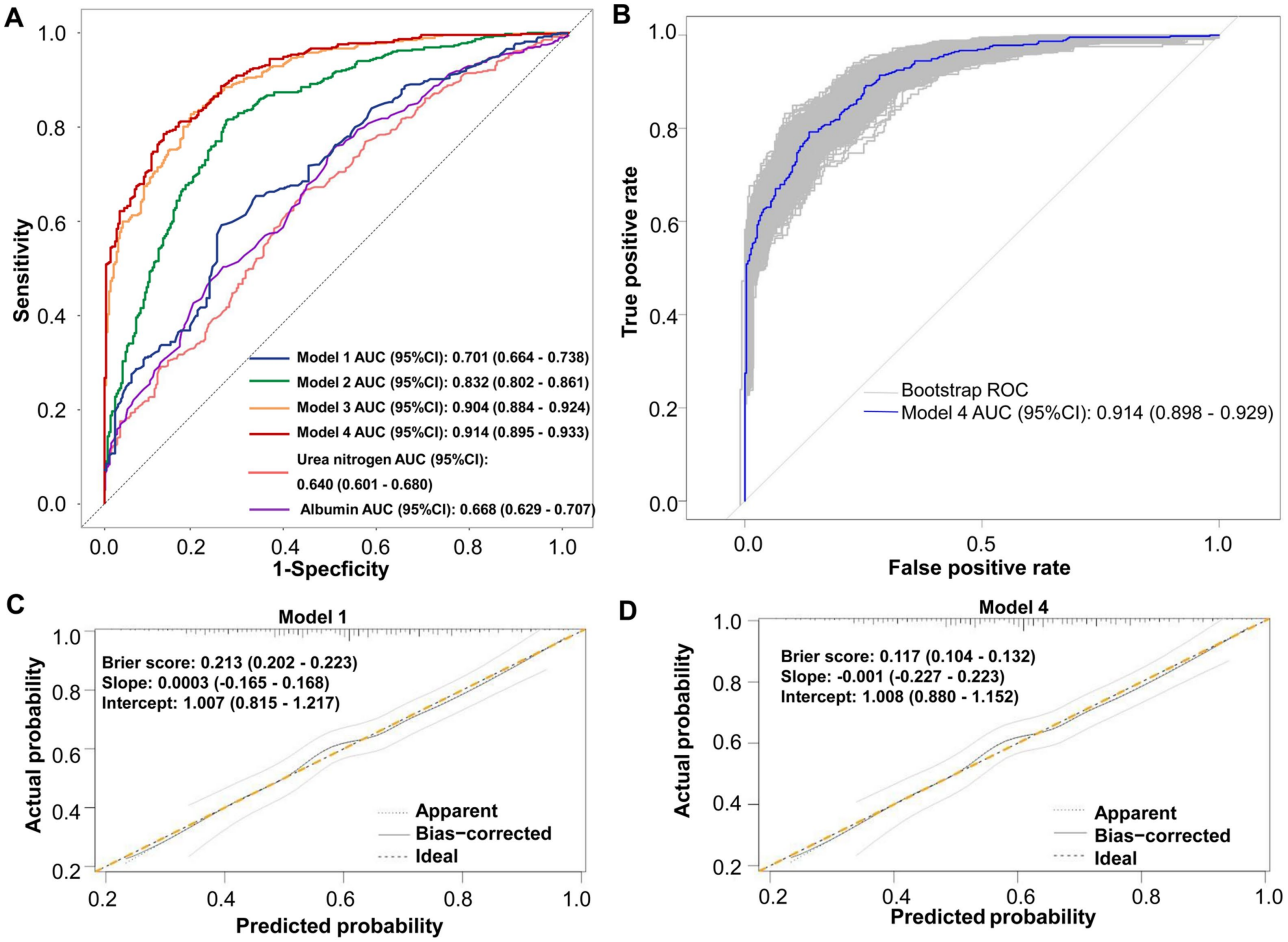
Discriminative performance and validation of models for CSVD. **(A)** ROC curves comparing the discriminative ability of multivariate models (Models 1–4) for CSVD. **(B)** Internal validation of Model 4 using the bootstrap method with 1,000 replicates. **(C, D)** Calibration plots for **(C)** BAR alone (Model 1) and **(D)** the full multivariate model (Model 4). The solid line represents the observed versus predicted probability of CSVD, with the dashed diagonal line indicating perfect calibration.

ROC curves examined the performance of multivariate models 1–4 in discriminating CSVD. As illustrated in Figure 6A, Model 4 (95% CI: 0.895–0.933, *p* < 0.001) demonstrated an AUC of 0.914 for discriminating CSVD, which was superior to that of the other models (Model 1 (BAR) AUC: 0.701, 95% CI: 0.664–0.738, *p* < 0.001; Model 2 AUC: 0.832, 95% CI: 0.802–0.861, *p* < 0.001; Model 3 AUC: 0.904, 95% CI: 0.884–0.924, *p* < 0.001). Importantly, the AUC of Model 1 (BAR) was higher than that of BUN alone (AUC: 0.640, 95% CI: 0.601–0.680) or albumin alone (AUC: 0.668, 95% CI: 0.629–0.707). The optimal cutoff value for Model 4 was determined to be 0.561 based on Youden’s index. At this threshold, the model achieved a sensitivity of 83.6% (95% CI: 80.3–86.9%) and a specificity of 81.6% (95% CI: 77.3–86.1%). For comparison, the Model 1 (BAR) achieved a maximum Youden’s index of 0.339, with a sensitivity of 59.0% (95% CI: 54.3–63.6%) and specificity of 74.8% (95% CI: 69.8–79.7%). To address potential optimism, we employed the bootstrap method by resampling each subject in the cohort with equal probability and reanalyzing the model on 1,000 randomly generated replicates of the dataset. The results indicated that Model 4 achieved a mean AUC of 0.914 (95% CI: 0.898–0.929), demonstrating robust model performance (Figure 6B). The narrow width of the 95% CI (3.1%) further supported its high stability. Furthermore, we assessed model calibration. The calibration plots (Figures 6C,D) and metrics showed that Model 1 (BAR) had reasonable calibration (intercept = 1.007, slope = 0.0003, Brier score = 0.213), while Model 4 demonstrated excellent calibration (intercept = 1.008, slope = −0.001, Brier score = 0.117), indicating its predicted probabilities were highly accurate. Therefore, the integration of BAR and relevant factors in Model 4 demonstrates excellent discriminative capability for the occurrence of CSVD.

### 3.8 Sensitivity analyses assessing the robustness of the BAR-CSVD association against kidney function

To verify that the association between BAR and CSVD was not merely a reflection of underlying kidney dysfunction, three sensitivity analyses were performed (Table 5). These analyses were designed to exclude potential confounding by kidney dysfunction rather than to evaluate BAR as a renal biomarker. First, after excluding patients with eGFR <60 mL/min/1.73 m^2^, BAR remained independently associated with CSVD (OR = 1.75, 95% CI: 1.32–2.32, *p* < 0.001), WMH (OR = 1.56, 95% CI: 1.22–2.00, *p* < 0.001), and EPVS (OR = 2.14, 95% CI: 1.73–2.66, *p* < 0.001). Second, the 18 patients with mild kidney insufficiency (serum creatinine 1.5–2.0 mg/dL) who had been excluded from the main analysis were re-integrated to form an expanded cohort (*n* = 780). These patients were not part of the original analysis cohort (*n* = 762), which included only those with creatinine ≤ 1.5 mg/dL. The baseline characteristics of the expanded cohort were comparable to those of the original cohort (Supplementary Table S1). In this expanded cohort, BAR remained significantly associated with CSVD (OR = 1.43, 95% CI: 1.12–1.82, *p* = 0.004), WMH (OR = 1.28, 95% CI: 1.05–1.56, *p* = 0.015), and EPVS (OR = 1.62, 95% CI: 1.36–1.93, *p* < 0.001). Finally, after forcibly adjusting for eGFR in Model 4, the associations between BAR and CSVD (OR = 1.67, 95% CI: 1.26–2.21, *p* < 0.001), WMH (OR = 1.51, 95% CI: 1.18–1.93, *p* < 0.001), and EPVS (OR = 2.09, 95% CI: 1.69–2.58, *p* < 0.001) remained statistically significant and robust.

**Table 5:** Results of sensitivity analyses evaluating the association between BAR and CSVD and its imaging markers after adjustments for kidney function.

Sensitivity analysis	CSVD		WMH		EPVS	
	OR (95% CI)	*p*	OR (95% CI)	*p*	OR (95% CI)	*p*
Sensitivity analysis 1: excluding participants with eGFR <60 mL/min/1.73 m^2^	1.75 (1.32–2.32)	<0.001	1.56 (1.22–2.00)	<0.001	2.14 (1.73–2.66)	<0.001
Sensitivity analysis 2: re-including participants with mild kidney impairment (creatinine 1.5–2.0 mg/dL)	1.43 (1.12–1.82)	0.004	1.28 (1.05–1.56)	0.015	1.62 (1.36–1.93)	<0.001
Sensitivity analysis 3: adjusting for eGFR in the multivariate model	1.67 (1.26–2.21)	<0.001	1.51 (1.18–1.93)	<0.001	2.09 (1.69–2.58)	<0.001

All sensitivity analyses are adjusted for sex, age, body mass index, systolic blood pressure, diastolic blood pressure, smoking status, drinking history, history of hypertension, diabetes, coronary heart disease, and stroke, lymphocyte count, C-reactive protein, total cholesterol, low-density lipoprotein cholesterol, high-density lipoprotein cholesterol, fasting plasma glucose, glycosylated hemoglobin, and uric acid. Sensitivity analysis 3 further includes eGFR as a covariate to directly adjust for its potential confounding effect. BAR, Blood Urea Nitrogen to Albumin Ratio; CSVD, Cerebral Small Vessel Disease; WMH, White Matter Hyperintensities; EPVS, Enlarged Perivascular Spaces; OR, Odds Ratio; CI, Confidence Interval; eGFR, estimated Glomerular Filtration Rate.

## 4 Discussion

To the best of our knowledge, this is one of the first comprehensive studies to report a significant independent association between BAR and CSVD after rigorous adjustment for a wide array of potential confounders. Our findings position BAR as a robust and novel biomarker linked to the overall burden of CSVD and specifically to its ischemic markers, notably WMH and EPVS.

The first finding of this study is that BAR was independently correlated with CSVD, with its level showing a positive correlation with disease presence. A linear dose–response relationship was further observed with the burden of WMH and EPVS. This aligns with prior research identifying BAR as a risk factor for cerebrovascular diseases (34). The pathophysiological link between BAR and CSVD can be conceptualized through the interplay of kidney and cerebral small vessel health. The brain and kidneys share anatomical and physiological similarities as high-flow organs with extensive microvascular networks, making them jointly vulnerable to systemic vascular insults (35–37). This “cross-talk” is evidenced by shared pathogenic mechanisms: kidney dysfunction accelerates systemic arteriosclerosis (38), a hallmark of CSVD (39, 40). Pathologies such as hypertensive arteriolar remodeling (41, 42) and atherosclerosis-induced impairment of the perivascular waste-clearance system contribute to EPVS formation (43, 44). Furthermore, kidney insufficiency can promote cerebral microvascular damage by disrupting cerebral blood flow autoregulation and compromising endothelial tight junctions (45). Thus, BAR, as a composite marker of kidney function and systemic inflammation, may capture this shared microvascular vulnerability, providing a plausible biological basis for its association with CSVD. Critically, our extensive sensitivity analyses confirmed that the association between BAR and CSVD remained robust after excluding participants with significant kidney impairment (eGFR < 60 mL/min/1.73 m^2^), including those with mild impairment (serum creatinine 1.5–2.0 mg/dL), or after forcibly adjusting for eGFR. This strengthens the contention that the BAR-CSVD relationship is not merely a proxy for advanced kidney disease but reflects a broader, integrative pathophysiological connection between kidney and cerebral small vessel health.

The second finding of this study was the lack of a significant association between BAR and lacunes, which contrasts with a previous report that identified BAR as a correlative factor (26). This null finding should be interpreted with caution, as it may reflect not only a true biological dissociation but also several methodological limitations. First, lacunes were treated as a binary variable (presence vs. absence), which reduces sensitivity and may obscure associations with lacune burden or severity. A semi-quantitative grading approach based on lacune count could be explored in future studies. Second, lacunes represent a heterogeneous pathology, ranging from incidental cavities to clinically significant infarcts, and such phenotypic variability may dilute any underlying association with BAR. Third, the cross-sectional, hospital-based design of this study limits mechanistic inference, and unmeasured confounders may further contribute to the null result. Despite these limitations, the null finding proved to be robust upon deeper investigation: it was not modified by the presence of hypertension, advanced age, or diabetes—the very factors most intimately tied to lacunar pathogenesis (46). This suggests that the biological pathway captured by BAR may be more specific to the processes underlying diffuse white matter damage (WMH and EPVS) rather than the focal cerebral infarction that gives rise to lacunes. This biological distinction is plausible: unlike the widespread parenchymal injury characterized by WMH and EPVS, lacunes often result from focal occlusive events in penetrating arterioles (47), and their association with systemic inflammatory markers has been less consistent (48–52). Nevertheless, given the methodological constraints acknowledged above, this interpretation remains tentative pending validation in prospective, community-based cohorts with more granular lacune phenotyping.

The third finding of this study indicated that BAR levels were significantly associated with the occurrence of CSVD in subgroup analyses, and this correlation remained consistent across most examined subgroups. However, this association was not statistically significant in specific subgroups, including those with higher BMI (≥24 kg/m^2^) or elevated FPG (≥6.1 mmol/L). This may be attributed to lower baseline CSVD risk and prevalence, which reduces the statistical power to detect an effect. Furthermore, in subgroups defined by potent risk factors such as diabetes (53), the dominant pathophysiological impact of these conditions on CSVD may overshadow the more modest contribution of BAR. Similarly, the complex relationship between obesity, kidney function, and albumin metabolism may confound the BAR-CSVD relationship in higher BMI subgroups (54). These observations suggest that the utility of BAR may be most evident in populations with a higher overall burden of vascular risk. The exploratory nature of these subgroup findings necessitates confirmation in larger, prospectively designed studies.

A key finding that justifies the use of BAR as a composite index is its superior discriminative performance compared to its individual components. While both BUN and albumin were correlated with CSVD in univariate analysis, the ratio provided a unique value. This is objectively demonstrated by our ROC analysis, where the AUC for BAR (0.701) was notably higher than that for BUN alone (0.640) or albumin alone (0.668). This indicates that BAR integrates the synergistic risk pathways of kidney/metabolic stress (via BUN) and systemic inflammation/endothelial vulnerability (via albumin) into a single, more powerful metric for identifying CSVD. The clinical utility of BAR lies in its unique profile as an accessible, low-cost, and pathophysiologically integrative biomarker. It not only offers a potential pragmatic triage tool for prioritizing MRI referrals in resource-limited settings but also provides complementary information on kidney and inflammatory pathways that refine risk assessment beyond conventional factors, all while maintaining operational simplicity through its derivation from two routine tests, enabling seamless integration into clinical workflows at “zero marginal cost.” Notably, Model 4 exhibited the highest discriminative efficiency, achieving an AUC of 0.914. Furthermore, the bootstrap test and calibration curves confirm the reliability of the model’s performance. Therefore, integrating BAR into clinical practice could enhance the accuracy of discriminating the severity of CSVD and inform intervention strategies.

Given the analysis of multiple related outcomes (the composite CSVD and its three individual imaging markers) and the performance of subgroup analyses, the issue of multiple testing was considered. A formal correction (e.g., Bonferroni) was not applied for the following reasons. First, the analysis of individual imaging markers was secondary and complementary to the pre-specified primary analysis of the composite CSVD outcome, aimed at elucidating the specific pathological features underlying the main association. Second, subgroup analyses were explicitly exploratory and hypothesis-generating in nature. The *p*-values for all analyses are presented unadjusted, and the interpretation of results, particularly for subgroup analyses, emphasizes the effect sizes and clinical plausibility while acknowledging the increased risk of Type I error.

This study has several limitations. First, as a cross-sectional, retrospective, single-center study, it cannot establish causality. Additionally, the relatively modest sample size may limit statistical power and raise the potential for selection bias. Second, the generalizability of our findings is constrained by two key factors: (1) the exclusion of patients with advanced chronic kidney disease limits the applicability of our results to populations with severe kidney impairment. Although our sensitivity analyses support the robustness of the BAR-CSVD association, future studies are needed to establish the utility of BAR in cohorts with severe kidney impairment. (2) More importantly, our samples were drawn exclusively from a hospital-based neurology population, which may introduce spectrum bias. The prevalence and severity of CSVD, as well as BAR levels in such a setting, could differ substantially from those in community-based populations, limiting the direct generalizability of our findings to the general community. Factors such as regional variations in diet (which affects BUN), differences in genetic background, and heterogeneity in clinical protocols (e.g., MRI equipment and laboratory assays) may also influence BAR’s baseline levels and its association with CSVD. Future studies should prioritize the external validation of this model in prospective, multi-center cohorts that include community-based populations. Third, our study relied on a single measurement of BAR obtained at hospital admission. While this reflects a realistic clinical scenario, and we mitigated variability by using pre-treatment samples and applying strict exclusion criteria, it remains susceptible to short-term and acute physiological fluctuations rather than reflecting stable long-term chronic risk profiles. The reported associations might be underestimated due to non-differential misclassification. Future studies with serial, outpatient measurements are warranted to confirm our findings.

In this cross-sectional retrospective study, we observed an independent association between BAR and CSVD, along with its ischemic imaging markers such as WMH and EPVS. Despite the absence of a significant relationship with lacunes, the findings highlight the potential utility of BAR as an accessible and cost-effective candidate marker. Before BAR can be considered for clinical implementation, several critical steps are required to translate this finding from a research association to a validated clinical tool. These steps include: validation in large, prospective, multi-center cohorts to confirm its generalizability across diverse populations and healthcare settings; determination of context-specific and standardized cut-off values for risk stratification; and evaluation through interventional studies to assess whether BAR-guided management ultimately improves patient outcomes. By establishing this foundational evidence, BAR could, in the future, contribute to addressing the growing concerns surrounding CSVD by facilitating timely risk assessment, particularly in the aging population of China.

## Data Availability

The raw data supporting the conclusions of this article will be made available by the authors, without undue reservation.
