# Supplementary material for: Association of blood urea nitrogen to albumin ratio with cerebral small vessel disease and its ischemic imaging markers: a cross-sectional study
Source: Front Neurol. 2026 May 21;17:1763901. doi: 10.3389/fneur.2026.1763901 (PMC13235146; doi:10.3389/fneur.2026.1763901)
Supplement: Supplementary file 1 [file Table_1.docx]

Supplementary Material

# Supplementary Figures and Tables

## Supplementary Figures


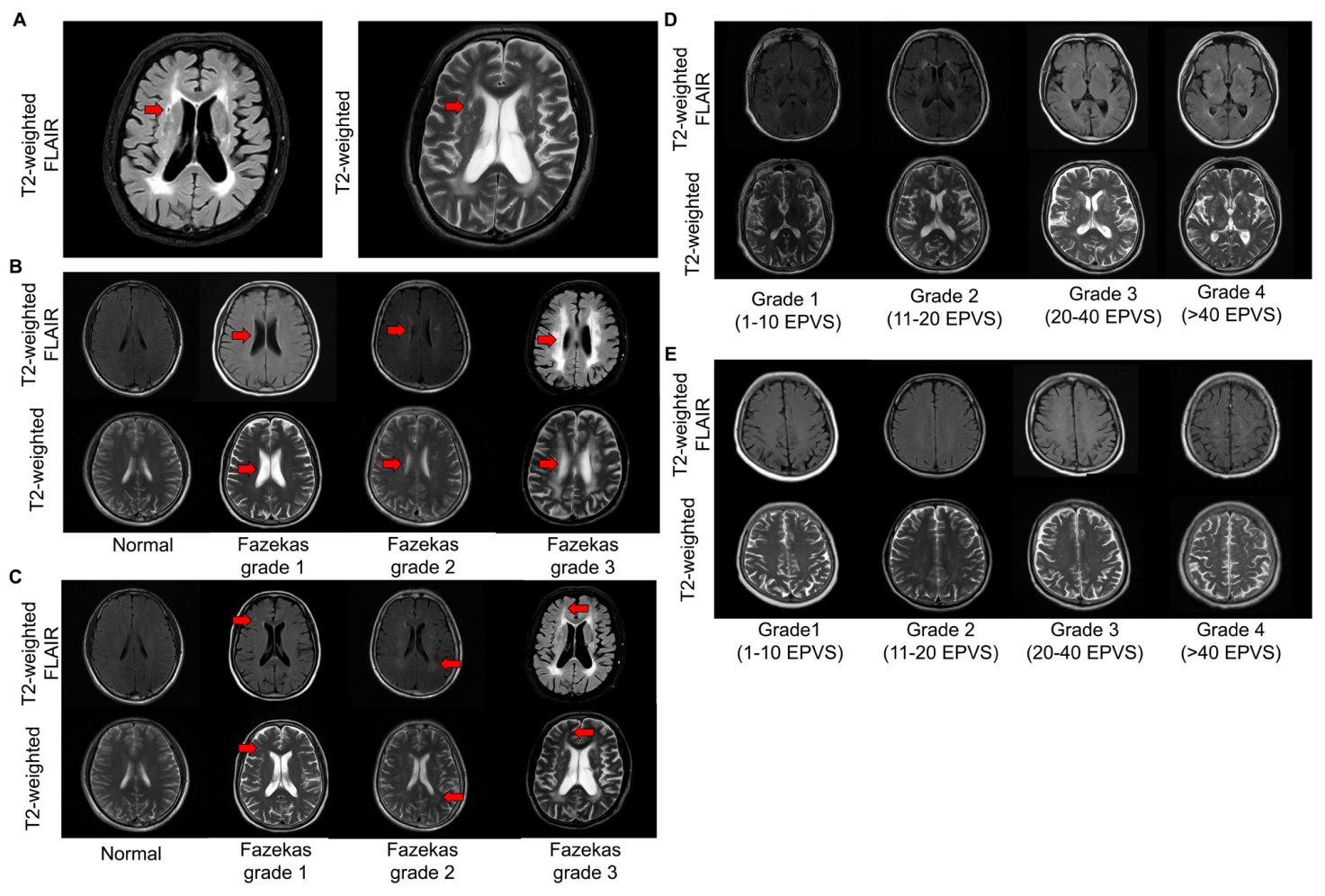


**Supplementary Figure S1.** Representative MRI examples of CSVD markers and their severity grading. (A) Lacunes. Example in the right periventricular white matter (arrows), demonstrating characteristic signal intensity across sequences. (B) PWMH. Representative images of Fazekas' grades 1, 2, and 3 (arrows). Detailed grading criteria are provided in the Methods section. (C) DWMH. Representative images of Fazekas' grades 1, 2, and 3 (arrows). Detailed grading criteria are provided in the Methods section. (D) EPVS in the basal ganglia. Representative images of visual grades 1 (mild) to 4 (severe). (E) EPVS in the centrum semiovale. Representative images of visual grades 1 (mild) to 4 (severe). All images are presented in pairs of T2-weighted FLAIR and T2WI. Abbreviations: CSVD, cerebral small vessel disease; PWMH, periventricular white matter hyperintensities; DWMH, deep white matter hyperintensities; EPVS, enlarged perivascular spaces; FLAIR, fluid-attenuated inversion recovery; T2WI, T2-weighted imaging.

## Supplementary Table S1. Comparison of baseline characteristics between the original main analysis cohort (n = 762) and the expanded sensitivity analysis cohort (n = 780).

| Variables | Original cohort (n = 762) | Expanded cohort (n = 780) | *P* |
| --- | --- | --- | --- |
| Age (years) | 63.00 (52.00, 70.00) | 63.00 (52.00, 70.00) | 0.894 |
| Male (%) | 403 (52.89) | 421 (53.97) | 0.827 |
| BMI (kg/m²) | 23.48 (21.59, 24.91) | 23.53 (21.64, 24.98) | 0.704 |
| SBP (mmHg) | 138.00 (123.00, 156.00) | 138.00 (123.75,156.00) | 0.910 |
| DBP (mmHg) | 84.00 (76.00, 95.00) | 84.00 (76.00, 95.00) | 0.862 |
| Smoking, n(%) | 218 (28.22) | 224 (28.72) | 0.827 |
| Drinking, n(%) | 93 (12.20) | 99 (12.69) | 0.772 |
| Hypertension, n (%) | 380 (49.87) | 394 (50.51) | 0.800 |
| Diabetes, n(%) | 114 (14.96) | 120 (15.38) | 0.817 |
| History of CHD, n(%) | 55 (7.22) | 57 (7.31) | 0.946 |
| History of stroke, n(%) | 104 (13.65) | 110 (14.10) | 0.796 |
| WBC (10^9^/L) | 7.14 (5.79, 8.67) | 7.17 (5.80, 8.70) | 0.823 |
| Neutrophil count (10^9^/L) | 4.30 (3.30, 5.92) | 4.34 (3.32, 5.99) | 0.778 |
| Lymphocyte count (10^9^/L) | 1.78 (1.40, 2.25) | 1.78 (1.40, 2.24) | 0.846 |
| CRP (mg/L) | 1.72 (0.50, 4.07) | 1.80 (0.50, 4.10) | 0.814 |
| TC (mmol/L) | 4.88 (4.13, 5.70) | 4.87 (4.10, 5.70) | 0.864 |
| LDL-C (mmol/L) | 3.12 (2.51, 3.76) | 3.10 (2.50, 3.76) | 0.913 |
| HDL-C (mmol/L) | 1.13 (0.98, 1.36) | 1.13 (0.97, 1.36) | 0.730 |
| FPG (mmol/L) | 5.21 (4.75, 5.93) | 5.21 (4.75, 5.95) | 0.942 |
| HbA1c (%) | 5.80 (5.54, 6.30) | 5.80 (5.60, 6.30) | 0.827 |
| eGFR (mL/min × 1.73 m^2^) | 94.82 (82.01, 105.81) | 93.92 (80.80, 105.50) | 0.435 |
| Uric acid (μmol/L) | 333.00 (274.00,404.75) | 335.00 (275.00,408.00) | 0.685 |
| Creatinine (μmol/L) | 67.60 (57.40, 81.30) | 68.20 (57.52, 82.12) | 0.433 |
| Blood urea nitrogen (mg/dL) | 13.58 (11.42, 15.98) | 13.64 (11.40, 16.12) | 0.823 |
| Albumin (g/dL) | 3.95 ± 0.36 | 3.94 ± 0.37 | 0.804 |
| BAR | 3.49 (2.93, 4.12) | 3.50 (2.93, 4.14) | 0.788 |

Note: CSVD, cerebral small vessel disease; BMI, body mass index; SBP, systolic blood pressure; DBP, diastolic blood pressure; CHD, coronary heart disease history; WBC, white blood cell; CRP, C-reactive protein; TC, total cholesterol; LDL-C, low-density lipoprotein cholesterol; HDL-C, high-density lipoprotein cholesterol; FPG, fasting plasma glucose; HbA1c, glycosylated hemoglobin; eGFR, estimated glomerular filtration rate; BAR, Blood urea nitrogen to serum albumin ratio.
